# Exercise-Induced Adaptations to Adipose Tissue Thermogenesis

**DOI:** 10.3389/fendo.2020.00270

**Published:** 2020-04-29

**Authors:** Pablo Vidal, Kristin I. Stanford

**Affiliations:** Department of Physiology and Cell Biology, Dorothy M. Davis Heart and Lung Research Institute, The Ohio State University Wexner Medical Center, Columbus, OH, United States

**Keywords:** exercise, obesity, white adipose tissue (WAT), brown adipose tissue (BAT), thermogenesis

## Abstract

Exercise training results in beneficial adaptations to numerous tissues and offers protection against metabolic disorders including obesity and type 2 diabetes. Multiple studies have indicated that both white (WAT) and brown (BAT) adipose tissue may play an important role to mediate the beneficial effects of exercise. Studies from both rodents and humans have identified exercise-induced changes in WAT including increased mitochondrial activity and glucose uptake, an altered endocrine profile, and in rodents, a beiging of the WAT. Studies investigating the effects of exercise on BAT have resulted in conflicting data in terms of mitochondrial activity, glucose uptake, and thermogenic activity in rodents and humans, and remain an important area of investigation. This review discusses the exercise-induced adaptations to white and brown adipose tissue, distinguishing important differences between rodents and humans and highlighting the latest studies in the field and their implications.

## Introduction

Exercise training is an important non-pharmacological strategy to prevent and treat metabolic diseases, including obesity and type 2 diabetes. Exercise results in adaptations to almost all tissues in the body that contribute to the beneficial effects of exercise to improve whole-body metabolic health. A single bout of moderate intensity exercise has dramatic effects on glucose metabolism, lowering circulating insulin concentrations and improving skeletal muscle insulin sensitivity (1). Exercise training, defined as repeated bouts of exercise over a period of weeks, months, or years can decrease insulin concentrations and improve glucose tolerance (1, 2).

While it is well-established that exercise induces adaptations to skeletal muscle (2) and the cardiovascular system (3), several studies have now determined that exercise also results in adaptations to adipose tissue that improve whole-body metabolic health (4–15). These exercise-induced adaptations to adipose tissue include increased mitochondrial activity (5, 10), decreased cell size and lipid content (11), reduced inflammation (12, 13), and, in rodents, increased presence of thermogenic brown-like adipocytes or “beige” cells (6, 10, 15). Exercise also alters the endocrine profile of adipose tissue, inducing the release of adipokines and lipokines that mediate tissue-to-tissue communication and contribute to the improved metabolic homeostasis seen with exercise (9, 14, 16). Here, we will discuss studies investigating the exercise-induced adaptations to white and brown adipose tissue in humans and rodents, with a particular focus on the adaptations that contribute to thermogenesis.

## Adipose Tissue

Adipose tissue is a type of connective tissue consisting primarily of mature adipocytes (~65–90% in volume) (17, 18), a cell type whose defining characteristic is accumulation of internal fat droplets (19). In addition to the mature adipocytes, adipose tissue consists of a stromal vascular fraction (SVF). The SVF is immensely heterogeneous, containing pre-adipocytes, mesenchymal stem cells, endothelial cells, and a variety of immune cells, including macrophages and natural killer T cells (20). The SVF is very dynamic and can respond and adapt to stimulus such as β-adrenergic stimulation (20) and exercise (8).

Adipose tissue can be broadly classified into two different types, white adipose tissue and brown adipose tissue (21). Certain stimuli such as cold, sympathetic activation (22), exercise (6, 23) or an enriched environment (24) can give rise to a third type of adipocytes, beige adipocytes, within the WAT.

### White Adipose Tissue

White adipose tissue (WAT) is composed of white adipocytes and its primary function is energy storage. Energy is stored by mature adipocytes in the form of triglycerides as one unilocular lipid droplet which occupies most of the cell volume and can vary in size (25). Adipose tissue is very dynamic, it can expand in size via hyperplasia or hypertrophy of the adipocytes (26, 27). WAT can be further subdivided into two different depots with distinct functions based on anatomical location, subcutaneous and visceral WAT (28).

### Subcutaneous WAT

Subcutaneous WAT (scWAT) is located beneath the skin. In mice, scWAT is located in the inguinal, anterior axillary and interscapular regions (28–30). In humans, scWAT locations can be divided into lower-body, comprising gluteal and leg depots, and upper-body, in the anterior abdominal wall region (28). These distinct locations of scWAT adapt differently to the same stimulus (26, 31). Under obesogenic conditions, lower-body adipocytes tend to expand via hyperplasia, which has been associated with improved metabolic adaptations (32), while upper-body adipocytes expand via hypertrophy (26). Increases in upper-body scWAT are correlated with decreased insulin sensitivity and impaired glucose tolerance (31).

### Visceral WAT

Visceral WAT (vWAT) surrounds internal organs. In mice, vWAT is found in the perigonadal, mesenteric, perirenal, retroperitoneal, cardiac, and triceps-associated regions (8, 28–30). In humans, vWAT is located in the intraabdominal (omental and mesenteric) as well as in the cardiac region (28). In lean individuals, vWAT accounts for 10–20% of the total fat mass in males and 5–8% in females (33).

There are distinct differences between scWAT and vWAT. These two adipose tissue depots behave and adapt differently to the same stimuli (26, 28, 34). Adipocytes in scWAT are smaller, have higher avidity for free fatty acid and triglyceride uptake, and are more sensitive to insulin compared to adipocytes from the vWAT (33, 35). Subcutaneous WAT has elevated expression of genes involved in glucose and lipid metabolism, and insulin signaling, compared to vWAT (36). Conversely, increases in vWAT are correlated with impaired glucose tolerance and increased insulin resistance (31) while increases in scWAT are correlated with improved metabolism (37).

### Brown Adipose Tissue

Brown adipose tissue (BAT) is a metabolically active tissue that burns carbohydrates and lipids to generate heat (38–40). Brown adipocytes are characterized by multilocular lipid droplets, a central nucleus and a high density of mitochondria (41, 42). The most distinctive feature of brown adipocytes is the high expression of the thermogenic protein uncoupling protein 1 (UCP1) (43). UCP1 is located in the inner membrane of mitochondria and uncouples the proton gradient potential generated by the electron transport chain. Release of this chemical gradient results in the dissipation of energy in the form of heat. In rodents, BAT is found in the interscapular, mediastinal, perirenal, axillary, and cervical regions (29, 30, 44). BAT is a mammal-specific tissue and in humans, it was long thought to be present only in infants. In 2009, multiple studies demonstrated that BAT is also present in adult individuals (45–48). In humans, BAT is found in the cervical, supraclavicular, axillary, and paravertebral regions (45, 49), as well as in the perirenal region in infants (50). Perirenal BAT consists mainly of dormant brown adipocytes that can be stimulated to give rise to active brown adipocytes (51). Brown adipose tissue mass is negatively correlated with BMI and age in humans (45). Given this, and the functional role of BAT, targeting BAT as a therapeutic to combat obesity and metabolic disorders has become increasingly important.

### Beige Adipocytes

Beige or brite (brown in white) adipocytes are a particular type of adipocytes within scWAT. Over 100 different stimuli are known to induce beiging, and most of them act through activation of the sympathetic nervous system (SNS) (52). Beige adipocytes have multilocular lipid droplets, a central nucleus, and a high density of mitochondria, similar to brown adipocytes. However, while brown adipocytes arise from *Pax7* and *Myf5* positive cells (53, 54), beige adipocytes arise from *Myf5* negative adipogenic stem cells within the adipose tissue (55, 56). White adipocyte tissue that has undergone beiging can be distinguished by the specific beiging markers *CD137, TBX1*, and *TMEM26* (30). Beige adipocytes function similarly to brown adipocytes in that they directly generate energy in the form of heat, contributing to thermogenesis. Beige adipocytes deviate from brown adipocytes in that they have a high degree of plasticity. In the absence of beiging stimuli, UCP1 expression, and mitochondrial content of beige adipocytes decrease and beige adipocytes transition to a white adipocyte phenotype (49). Increasing beige adipocytes has significant potential to combat obesity and type 2 diabetes.

## Exercise-Induced Adaptations to WAT

**Figure 1:**
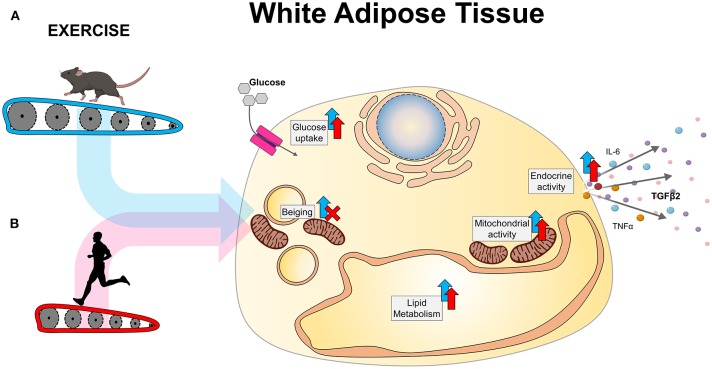
Exercise-induced adaptations to WAT in **(A)** rodents and **(B)** humans.

Exercise is an important therapeutic to prevent and treat metabolic diseases, including obesity and type 2 diabetes. Exercise results in adaptations to almost all tissues in the body, including adipose tissue. Exercise increases whole-body energy expenditure as chemical energy is converted into kinetic energy. During acute exercise, WAT has an important role in supplying this additional energy requirement from the triglyceride stores within the mature adipocytes (57). Independent from its role during acute exercise, chronic exercise leads to several metabolic adaptations in WAT (Figure 1). In this section, we will be reviewing the different metabolic adaptations that occur in WAT with exercise in both rodents and humans, including thermogenesis, mitochondrial adaptations, glucose metabolism, lipid metabolism, and endocrine adaptations.

### Thermogenic Adaptations to WAT

An important exercise-induced adaptation to scWAT in rodents is the beiging of scWAT. Exercise induces an upregulation of thermogenic genes such as *Prdm16* and *Ucp1* in inguinal scWAT (6, 15, 58, 59) and an increased presence of adipocytes with multilocular lipid droplets (6, 60). The appearance of beige adipocytes does not occur homogeneously, as some regions of the inguinal scWAT are more prone to beiging than others (58, 61). This exercise-induced beiging is specific to scWAT, in particular the inguinal scWAT (8), and does not occur in vWAT (23, 60, 62). Beiging of scWAT is the molecular mechanism that leads to increased thermogenesis in WAT with exercise, as beige adipocytes increase non-shivering thermogenesis.

While beiging is an important adaptation to exercise, it is unclear why exercise induces a beiging of scWAT. Beiging of scWAT by non-exercise stimuli, including through cold-exposure, environmental factors or pharmaceuticals, is thought to be induced through a heat compensatory mechanism in which adrenergic stimulation compensates for heat loss with the upregulation of *UCP1* (44, 63–65). This explanation does not make sense in the context of exercise-induced beiging, because exercise itself increases heat production (66, 67). Several hypotheses have been proposed as the underlying mechanism, one of which is an increase in sympathetic innervation, which occurs in scWAT during exercise (52, 68) Other hypotheses have indicated that beiging occurs in response to the exercise-induced release of myokines, such as irisin (23), myostatin (69), meteorin-like 1 (Metrnl) (70), lactate (71), and β-aminoisobutyric acid (BAIBA) (72), or other secreted factors released during exercise, including brain-derived neurotrophic factor (BDNF) (24). More investigation is needed to fully understand this complex mechanism. These hypotheses are all important and plausible, but the most likely explanation is that the exercise-induced beiging of scWAT occurs because exercise decreases the adipocyte size and lipid content in scWAT, decreasing insulation of the body and necessitating heat production, which results in the beiging of scWAT (52, 73). The fact that mice are commonly housed at 20–22°C, the habitual indoor temperatures for humans, which itself contributes to mice being under chronic cold stress (74), provides further support for this explanation.

To address the hypothesis that beiging occurs in response to a loss of fat mass in a cold stress environment, multiple studies have investigated the effects of exercise at thermoneutrality (30°C) (14, 75, 76). Interestingly, when mice are housed at thermoneutral conditions, the exercise-induced increase of thermogenic gene expression and appearance of multilocular adipocytes exercise is blunted in male and female mice (75, 76), and this occurred independent of changes in body mass, fat mass, or running distance. Interestingly, one of these studies investigated female mice and determined that total running distance was lower at thermoneutrality (~40%) (76) and observed no differences in body weight or adiposity compared to sedentary mice, while another study determined that running distance was increased at thermoneutrality in male mice (~50%) compared to mice at room temperature (75). These mice also had lower body mass compared to sedentary mice and mice housed at room temperature. While these discrepancies make some of the nuances between these studies difficult to interpret, each study determined that exercise-induced increase in thermogenic genes was blunted at thermoneutrality. These data suggest that the exercise-induced beiging is not a direct consequence of exercise, it is indirectly induced through other stimuli such as increased cold stress due to loss of WAT mass.

Several human studies have determined that exercise in humans does not induce beiging of scWAT (77–80). In lean or obese individuals, 10–16 weeks of endurance training did not change the expression of thermogenic genes including *UCP1, PRDM16*, and *PGC1A* in scWAT in males and females (77, 80–82). Studies conducted in highly exercise-trained populations and individuals with a more active lifestyle have also not observed any differences in *UCP1* expression in scWAT compared to sedentary controls (83, 84). These results collectively indicate that exercise does not induce beiging in humans.

The mechanistic reason as to why rodents and humans have opposite thermogenic adaptations in WAT is currently unknown. Similar to what has been discussed earlier, it is likely a result of cold stress; since rodents are smaller, they have a higher surface to volume ratio that makes them more susceptible to cold stress. Exercise decreases WAT accumulation, increasing cold stress, and thermogenic adaptations are increased to counter this effect. This would not be the case in humans, so the loss of WAT may not induce the same thermogenic response. However, most human studies investigating the effects of exercise on WAT have been conducted indoors in controlled environments. Investigating human subjects who exercise in the cold (i.e., skiers, open water swimmers) might result in a thermogenic response to human WAT.

### Mitochondrial Adaptations to WAT

Exercise increases mitochondrial activity and density in scWAT and vWAT in rodents (5–8, 10, 58, 60, 85–87). Eleven days of voluntary wheel cage running increases the oxygen consumption rate of scWAT (6) and upregulates mitochondrial genes in both scWAT (6, 86) and vWAT (7, 8, 10, 58, 85). Importantly, exercise at thermoneutrality also results in upregulation of electron transport chain proteins (76), indicating that the increase in mitochondrial activity after exercise is independent of the beiging of WAT. *In vitro* studies indicate that exercise increases basal oxygen consumption rate of adipocytes differentiated from the SVF of scWAT (inguinal) or vWAT (perigonadal) of exercised mice (8), however maximal respiratory capacity only increased in adipocytes derived from scWAT (8). These data indicate that mitochondrial adaptations with exercise occur in both scWAT and vWAT in rodents, independent of beiging.

Exercise induces mitochondrial adaptations in human scWAT in lean male subjects (83, 88, 89) or young obese female subjects (77). Six weeks of high-intensity interval training (HIIT) increased mitochondrial respiration of scWAT (88). Ten to eighteen sessions of alternating continuous moderate-intensity training and HIIT did not change expression of genes involved in oxidative phosphorylation such as *PGC1A* or *COXIV* (78, 83, 90), but long term aerobic exercise-training increased expression of several genes involved in oxidative phosphorylation (89) and mitochondrial biogenesis (83). Exercise induced mitochondrial adaptations in vWAT have not been investigated in humans. Together these data indicate that exercise or increased physical activity increases mitochondrial activity in mouse and human WAT.

### Adaptations to Glucose Metabolism in WAT

Exercise improves whole-body glucose homeostasis in rodents (91) and humans (1). Exercise increases glucose uptake and insulin sensitivity of scWAT (6, 15) and induces upregulation of genes and proteins involved in glucose metabolism in scWAT and vWAT (7, 8). These data indicate that exercise improves glucose metabolism in WAT in rodents. Here, we will focus on the effects of exercise in glucose homeostasis in WAT.

Recent studies have investigated the effects of exercise at thermoneutrality on glucose metabolism, with conflicting results. One study found that exercise still resulted in improvements in whole-body glucose tolerance (75), whereas another found no effect of exercise on whole-body glucose homeostasis at thermoneutrality (76). Interestingly, the latter found that there was an increase in *in vivo* insulin-stimulated ^3^H-2DG uptake in vWAT at thermoneutrality, but no changes were found in scWAT (76). In the latter study, the lack of exercise-induced changes to glucose metabolism can likely be attributed to the fact that mice at thermoneutrality ran ~40% less than mice at room temperature (76). As the results from these two studies are conflicting, the effects of exercise on glucose metabolism at thermoneutrality are unclear. Further research is essential to elucidate the effects of exercise at thermoneutrality on glucose metabolism and determine which adaptations arise at a systemic level and which are specific to the WAT.

Studies investigating exercise-induced adaptations to glucose homeostasis in human WAT are less comprehensive. One study determined that 6 months of exercise upregulated genes involved in glucose metabolism in lower-body scWAT (89). Two weeks of exercise increased insulin-stimulated glucose uptake in lower-body scWAT, but not upper-body scWAT or vWAT (92). These data indicate that scWAT and vWAT, and even upper-body and lower-body scWAT, have distinct adaptations to glucose metabolism with exercise. This is of particular interest to human physiology as humans with a higher proportion of upper-body WAT have been correlated with impaired glucose tolerance, while humans with a higher proportion of lower-body WAT are associated with improved glucose levels (32). These data indicate the lower scWAT has a prominent role on the effect on whole-body glucose homeostasis and is more susceptible to exercise.

### Adaptations to Lipid Metabolism in WAT

Exercise effects lipid metabolism in WAT during exercise. Moderate exercise (40–65% VO_2_ max) acutely increases whole-body lipolysis two to three times over basal rates after exercising for 30 min, and increases lipolysis up to 5-fold over basal after 4 h of exercise (93). Here, we will focus on the chronic adaptations of exercise to WAT with regard to lipid metabolism.

In rodents, exercise induces several adaptations that affect lipid metabolism including changes in gene expression (6, 8, 94), post-translational modifications (7) and an altered lipidomic profile (94). Two to three weeks of voluntary wheel cage running upregulates genes involved in fatty acid oxidation in scWAT and vWAT (6, 8), and genes involved in phospholipid metabolism in scWAT (94). Twelve days of voluntary wheel cage exercise increases phosphorylation of hormone sensitive lipase (HSL) (86), and exercise over a longer duration (6 weeks) increases phosphorylation of adipose triglyceride lipase (ATGL) (7). These post-translational modifications result in increased lipolytic activity of ATGL and HSL (95–97). Another study demonstrated that chronic treadmill training (8 weeks) did not increase the rate of lipolysis in isolated adipocytes under basal conditions, but when these adipocytes were stimulated by a β-adrenergic agonist, lipolysis was significantly increased in adipocytes isolated from exercised mice compared to adipocytes isolated from sedentary mice (98). Together, these results suggest that exercise induces adaptations that increase lipolysis.

Exercise also induces extensive adaptations to the lipidomic profile of scWAT in rodents. Previous work in our laboratory demonstrated that 3 weeks of exercise dramatically alters the lipidome of scWAT. Exercise significantly decreased the overall abundance of triacylglycerol (TAG), phosphatidylserines (PS) lysophosphatidylglycerols and lysophosphatidylinositols (LPI) (94). In addition to the changes in overall lipid classes, there were also decreases in several specific molecular species of phosphatidic acid, phosphatidylethanolamines (PE), and PS. These changes corresponded with a significant upregulation of several genes involved in phospholipid metabolism. These data suggest molecular species-specific remodeling of phospholipids and TAGs in scWAT in response to exercise (66, 94). The functional consequence of the exercise-induced changes to the lipidome of scWAT have not been identified, but that will be the focus of future investigation.

Research on the effects of chronic exercise on lipid metabolism in humans has not been thoroughly investigated. Studies have shown that active individuals (self-reported exercise >3x per week) have increased levels of *CPT1B*, the rate-limiting enzyme in fatty acid oxidation, in scWAT compared to sedentary individuals (83), and 6 months of exercise upregulates several genes involved in lipid metabolism (89). These data indicate that long-term exercise increases fatty acid oxidation in human WAT. However, shorter duration exercise interventions do not alter adaptations to lipid metabolism in WAT (82, 83). Three weeks of exercise in sedentary individuals did not change *CPT1B* levels (83), and 12 weeks of exercise in obese subjects did not change expression levels of *ATGL, HSL*, or other lipolytic enzymes (82). Taken together, these data indicate that exercise upregulates lipid metabolism in WAT of both rodents and humans.

### Endocrine Adaptations to WAT

Exercise induces considerable adaptations to the secretory profile of several tissues, including adipose tissue (13, 99). Secretory factors released from adipose tissue have been labeled as adipokines. Four or more weeks of exercise in rodents decreases leptin and adiponectin mRNA levels in scWAT (100) and circulation (87, 100, 101) in rodents and humans. Exercise also increases expression of other factors such as TNF-α and IL-6 in both WAT depots and in circulation (85, 100).

Recent work in our laboratory determined that transplantation of scWAT from exercised donor mice into sedentary recipient mice resulted in improved whole-body glucose tolerance. Glucose uptake was also increased in BAT, soleus and tibialis anterior, indicating that an endocrine factor is released from exercise-trained scWAT to mediate these effects (6). TGF-β2 was recently identified as the adipokine responsible for these beneficial effects on glucose metabolism (14). TGF-β2 is an adipokine secreted in response to exercise in both rodents and humans from WAT. In rodents, acute treatment with TGF-β2 increased glucose uptake in soleus, heart and BAT, and increased fatty acid uptake in skeletal muscle. Notably, adipose tissue specific TGF-β2 knockout mice did not have exercise-induced improvements in systemic glucose uptake (14).

Exercise can also induce adaptations in WAT through myokines such as myostatin and BAIBA. Myostatin is a well-known factor that inhibits skeletal muscle growth (102). Exercise decreases myostatin levels in skeletal muscle and serum (103). Reduced levels of myostatin promote beiging of the scWAT in rodents (104) and are correlated with improved insulin sensitivity in humans (103). During exercise, increase in PGC1α triggers the secretion of β-aminoisobutyric acid (BAIBA) in both rodents and humans. BAIBA promotes beiging of scWAT in rodents while it is inversely correlated with serum glucose and insulin levels in humans (72). These data indicate that exercise stimulates release of secretory factors, from WAT as well as other tissues like skeletal muscle, that result in positive metabolic systemic and WAT specific adaptations.

### Effects of Endurance vs. Resistance Exercise on WAT

Exercise can be broadly divided into endurance (aerobic) and resistance (strength) training (2). There have been several studies investigating the different adaptations of endurance and resistance training in skeletal muscle (2, 105), but this is not the case with adipose tissue. Most studies have investigated the effects of endurance training on adipose tissue, using treadmill or voluntary wheel cage running in rodents, and running or cycling for human studies. Some studies have compared the effects of different intensities, moderate (MIT) or high-intensity (HIT) endurance training on adipose tissue and found that MIT and HIT had similar effects on WAT in rodents (106, 107) and humans (92, 108). Meta-analysis comparing the effect of MIT or HIT on adiposity in humans found HIT resulted in a greater decrease in total fat mass (109). A few human studies have mixed endurance and resistance training in their exercise protocols, without finding any striking differences when compared to just endurance training (14, 77, 79, 82). However, to our knowledge, the direct effect of resistance compared to endurance exercise in adipose tissue has not been investigated.

## Exercise-Induced Adaptations to BAT

**Figure 2:**
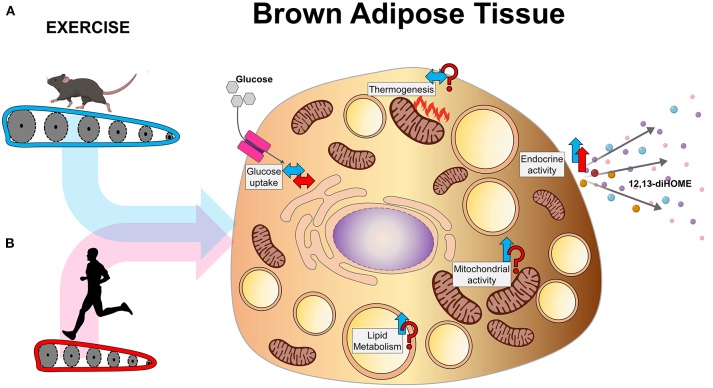
Exercise-induced adaptations to BAT in **(A)** rodents and **(B)** humans.

BAT accounts for a small percentage of total fat mass than WAT, but it is a much more metabolically active tissue than WAT (110). Exercise increases energy expenditure, thus indirectly increasing in thermogenesis (111). BAT and WAT functions are different, and so are their exercise-induced adaptations. Here, we will discuss the different metabolic adaptations that occur in BAT with exercise in both rodents and humans (Figure 2).

### Thermogenic Adaptations to BAT

The thermogenic effects of exercise on BAT in rodents have been thoroughly investigated, with conflicting results. Eleven weeks of swimming (6 days/week; 2 h per day) increased blood flow and oxygen consumption in response to acute injection with norepinephrine (NE) (112, 113), indicating that exercise may increase sensitivity to adrenergic stimulation in BAT. These data are difficult to interpret because swimming as an exercise modality indirectly results in cold stress. Interestingly, these studies found that when the water temperature is 32, 36, or 38°C, acute injection of NE had the same response to increase blood flow and oxygen consumption, but BAT mass was only increased when the water temperature was 32°C (112). Other studies investigated the effects of exercise on BAT using 6 weeks of treadmill training as the exercise protocol (114). Interestingly, there was no effect of treadmill exercise to affect oxygen consumption or blood flow at rest or after NE injection (114, 115). Furthermore, BAT mass and protein content were decreased with 6 weeks of treadmill training (115, 116), regardless of the ambient temperature of the exercise (room temperature or 4°C) (116). In female rats, 6 weeks of treadmill exercise increased BAT mass and total protein content (117), but 9 weeks of treadmill training reduced BAT mass and decreased *UCP1* expression (118). The reason for this is unclear, but it is possible that the discrepancies between these two studies could be explained by differences in the rat strain studied, as the first study used Sprague-Dawley while the latter used F-344 NNia. These data indicate that different exercise modalities, or different animal strains, could result in different adaptations to BAT.

More recent studies have indicated that exercise does not affect, or even decreases, BAT activity (58, 86, 119). Twelve days of voluntary wheel cage running in mice did not alter BAT mass (86), and 6 weeks of treadmill training in rats did not affect BAT mass, brown adipocyte size or *Ucp1* expression (58, 119). Oxidation of palmitate was also reduced in BAT *ex vivo* after 6 weeks of treadmill training, indicating exercise decreases fatty acid oxidation in BAT (58). Exercise at thermoneutrality also reduced BAT mass and did not alter markers of thermogenesis (75). These data indicate that exercise does not increase thermogenic activity in BAT in rodents in the absence of a cold stress (i.e., swimming).

There is currently a paucity of data that has investigated the thermogenic adaptations of BAT with exercise in humans. Studies have determined that endurance trained athletes subjected to cold exposure have decreased glucose uptake in BAT compared to sedentary subjects (84, 120). It is important to note that the current gold standard to measure BAT activity in humans is ^18^FDG-PET/CT (121), and humans studies have only determined BAT mass and activity in the context of its ability to take up glucose. Moreover, cold exposure is frequently needed to activate BAT for detection by ^18^FDG-PET/CT scans. Other methods like infrared thermography (122) and T2 mapping (123) have been developed to evaluate BAT presence, but they have not yet been used to assess differences in BAT activity with exercise. Fat T2 relaxation time mapping is based on BAT having higher water content than WAT. This technique measures BAT activity and does not require cold exposure for detection (123). The use of these new techniques will be important to truly ascertain the effects of exercise on BAT in humans *in vivo*.

### Mitochondrial Adaptations to BAT

The effects of exercise on mitochondrial activity in BAT have also been investigated. In rodents, 2–8 weeks of exercise did not change or decreased expression of mitochondrial genes (8, 58, 75, 76). Recent work in our laboratory determined that 11 days of voluntary wheel cage running (VWR) in male mice decreased basal oxygen consumption rate (OCR) in brown adipocytes differentiated from the SVF of BAT (8), but cells from both sedentary and exercise-trained BAT were able to respond to pharmacological stimulation to a similar extent. Eleven days of VWR decreased NADH autofluorescence, an indirect marker of metabolism, compared to the sedentary controls (8). In contrast, 6–8 weeks of treadmill training in rats significantly increased expression of proteins involved in mitochondrial biogenesis, such as *PGC1*α, *NRF1*, or *TFAM* (119, 124). The reason for the discrepancies in these studies are unclear, although duration, exercise modality, or species investigated (rat or mouse), could contribute to these different responses to exercise.

Studies on the effect of exercise in BAT mitochondria in humans are limited. One study found no differences on *PGC1*α expression in BAT between endurance athletes and sedentary males (84). Overall, exercise appears to decrease mitochondrial activity in BAT in mice, but more human studies are needed to elucidate the effects of exercise on mitochondrial activity in BAT.

### Adaptations to Glucose Metabolism in BAT

The effects of exercise on glucose uptake in BAT in rodents are conflicting. On one hand, some studies have shown that 2–8 weeks of exercise upregulates expression of genes involved in insulin signaling, glucose and fatty acid oxidation in BAT (8, 124, 125). However, 2 weeks of exercise decreased basal glucose uptake in brown adipocytes differentiated from SVF (8). Another study indicated that 6 weeks of exercise did not effect *in vivo* glucose uptake in BAT at room temperature or thermoneutrality, measured by *in vivo* insulin-stimulated ^3^H-2DG uptake (76). These data reveal that, although exercise results in an upregulation of genes involved in glucose metabolism, *in vivo* data in rodents indicates that exercise does not increase glucose uptake in BAT.

Several studies have indicated that exercise does not alter glucose uptake in BAT in humans. As little as 6 sessions of HIIT or moderate-intensity exercise-training in a 2 week period decreased insulin-stimulated glucose uptake in BAT (92), and 6 weeks of moderate-intensity continuous training did not affect cold-stimulated glucose uptake measured by ^18^FDG-PET/CT (126). In addition, endurance athletes have reduced glucose uptake in BAT when subjected to cold stimulation compared to sedentary subjects (measured by ^18^FDG-PET/CT) (84). Another study determined that there was no association of BAT mass or activity to physical activity in a cohort of 130 healthy, sedentary subjects (127). These data indicate that exercise or increased physical activity does not increase glucose metabolism in human BAT.

### Adaptations to Lipid Metabolism in BAT

The effects of exercise on lipid metabolism in BAT has not been thoroughly investigated. Eleven days of exercise increased expression of genes involved in fatty acid oxidation (8), but decreased expression of genes involved in fatty acid biosynthesis (94), phospholipid metabolism (94) and lipolysis (8, 75). Oxidation of palmitate was also reduced in BAT *ex vivo* after 6 weeks of treadmill training (58).

Exercise affects the lipidomic profile of BAT by increasing total abundance of TAGs phosphatidylcholines (PC) and cholesterol esters, while decreasing cardiolipins and lysophosphatidylglycerols (94). Exercise also significantly increased several specific molecular species of PC and PE in BAT. These data show that exercise decreases lipid metabolism in BAT. To our knowledge, there are currently no studies analyzing the effect of exercise on lipid metabolism in human BAT. While it is clear that BAT lipid metabolism changes with exercise, the role of the exercise-induced decrease in lipolysis or changes in BAT lipidome have not been identified and will be the topic of future investigations.

### Endocrine Adaptations to BAT

It is important to note that in most cases, particularly in human studies, BAT activity, and mass are measured by glucose uptake. This is important in most settings, however, since exercise is a thermogenic activity it is unlikely that exercise would increase glucose uptake in BAT. This has led several groups to hypothesize that exercise may alter the endocrine activity of BAT. In fact, multiple studies have identified an endocrine role for BAT in response to exercise (13, 16, 128). Recent work in our laboratory identified the lipokine, 12,13-diHOME, to be released from BAT in response to exercise in mice and humans (9) Upregulation of 12,13-diHOME activates fatty acid uptake and oxidation in skeletal muscle without affecting glucose homeostasis (9). This data shows a direct role of BAT to improve metabolic health with exercise. These are the first data to identify a secreted factor from BAT with exercise to mediate skeletal muscle metabolic adaptations.

## Future Directions and Conclusions

Exercise results in positive metabolic adaptations in both white and brown adipose tissue. Exercise increases mitochondrial activity, glucose metabolism, and endocrine activity in WAT in both rodents and humans. Notably, beiging of WAT only occurs with exercise in rodents, but both humans and rodents have increased mitochondrial activity independent of beiging of WAT. Exercise increases endocrine activity of BAT but does not affect glucose uptake in rodents and humans. Additionally, exercise does not affect thermogenesis and decreases mitochondrial activity in BAT in rodents.

An important point of investigation has been the effects of exercise-induced beiging in WAT. While this adaptation has been clearly identified in rodents, studies in humans have not identified the same effects. More recent studies have begun to investigate the effects of exercise at thermoneutrality to parse apart the direct effects of exercise on beiging, and have demonstrated that exercise at thermoneutrality blunts the effects of exercise on thermogenic gene expression (75, 76). Expanding these studies will provide greater insight and translational relevance for determining the effects of exercise on WAT (and potentially BAT).

Most of the studies discussed in this review have been conducted in either males or females. This is of particular importance as there are clear sex differences in adipose tissue depots among males and females, with females having a higher percentage of WAT (27) and higher BAT activity at rest (45). Another important issue in the field of BAT thermogenesis, especially in human studies, is the measurement of BAT activity. ^18^FDG-PET/CT is the gold standard for measurement of BAT mass and activity in humans, however, this analysis is solely based on the ability of BAT to uptake glucose to use it as a substrate. This highlights the importance of new techniques to accurately measure BAT activity and establish *in vivo* measurements of BAT thermogenic capacity, including in the context of exercise. Newer techniques such as infrared thermography and T2 mapping are potential mechanisms to elucidate the adaptations of BAT to exercise.

There is a need for the comprehensive understanding of the mechanisms underlying the chronic adaptations of adipose tissue with exercise. A single session of exercise leads to acute changes in expression of several genes (129). Successive bouts of exercise most lead to a cumulative effect of these acute changes resulting in chronic adaptations, which contribute to changes in glucose metabolism, fatty acid metabolism, and mitochondrial activity. Post-translational modifications such as protein phosphorylation regulate protein activity (130), and chronic exercise increases overall phosphorylation of proteins such as HSL and ATGL, which result in increased lipolytic activity (7, 75). Epigenetic modifications may also be underlying drivers of exercise-induce adaptations to exercise; studies have shown that exercise results in changes to the genome-wide DNA methylation pattern of human WAT (131, 132). These studies indicate that epigenetic modifications could oversee the chronic adaptations to adipose tissue with exercise by promoting or inhibiting expression of metabolic genes. Understanding factors that trigger exercise-induced adaptations remains an open field that will be an important for future investigations.

Together these studies highlight the importance of exercise to alter function of WAT and BAT that could provide important targets to improve metabolic health and reduce obesity. Future studies will investigate other mechanisms by which exercise exerts metabolic adaptations on adipose tissue such as increased mitochondrial function, improved glucose homeostasis or endocrine function, providing important translational relevance for exercise as a therapeutic tool.

## Author Contributions

PV and KS designed, wrote, and edited the manuscript.

## Conflict of Interest

The authors declare that the research was conducted in the absence of any commercial or financial relationships that could be construed as a potential conflict of interest.
